# The psychological burden of diabetes: common mental health disorders, their impact on disease outcomes and strategies for intervention

**DOI:** 10.1007/s40200-026-01915-6

**Published:** 2026-03-06

**Authors:** Javed Latoo, Yasser Saeed Khan, Armaan Latoo, Majid Alabdulla, Farida Jan, Nasseer Masoodi, Daljit Sura, Shariful Islam, Ovais Wadoo

**Affiliations:** 1 Department of Psychiatry, Hamad Medical Corporation, Doha, Qatar; 2 College of Medicine, Qatar University, Doha, Qatar; 3 Barts and The London School of Medicine and Dentistry, Queen Mary University of London, London, UK; 4 Department of Psychiatry, Northamptonshire Healthcare NHS Foundation Trust, Northampton, UK; 5 Department of Medicine, Hamad Medical Corporation, Doha, Qatar; 6 North Street Medical Care, Romford, Greater London, UK; 7 Department of Nutrition, College of Health and Human Sciences, Texas Tech University, Lubbock, TX USA

## Abstract

Diabetes mellitus is associated with a high burden of mental health issues, including both formal psychiatric disorders, such as depression and anxiety, and diabetes-specific psychological challenges like diabetes-related distress, psychological insulin resistance, and fear of hypoglycemia. These mental health difficulties significantly impair self-management, treatment adherence, and quality of life, increasing the risk of complications and mortality. The paper reviews the bidirectional relationship between diabetes and mental disorders, driven by biological and behavioural mechanisms. It highlights the high prevalence of depression, anxiety, cognitive impairment, and eating disorders among people with diabetes. The consequences of untreated mental health conditions include poor glycemic control, greater healthcare costs, increased hospitalization, and lower health-related quality of life. The review presents a range of evidence-based management strategies, including early screening, psychological and pharmacological interventions, lifestyle changes, and integrated collaborative care models. Particular emphasis is placed on the role of physicians and endocrinologists in recognizing and managing psychological difficulties. The paper also discusses challenges in low-resource settings and underscores the need for culturally adapted accessible interventions. It argues for a paradigm shift towards integrated, person-centred care models that address both physical and mental health needs in diabetes, offering practical recommendations for clinicians and policymakers.

## Introduction

Diabetes mellitus, a chronic metabolic disorder characterised by hyperglycemia, has been linked to an increased risk of mental health problems [1, 2]. These include both formal psychiatric diagnoses such as depressive and anxiety disorders, as well as psychological challenges that are specific to living with diabetes [3]. The latter include Diabetes-related Distress (DRD), Psychological Insulin Resistance (PIR), and Fear of Hypoglycemia (FOH). It is important to note the distinction between clinically diagnosable psychiatric disorders and diabetes-specific psychological complications, which, while not formal diagnoses, can significantly affect diabetes management and overall wellbeing. DRD describes the negative feelings and burden associated with the constant self-care required by the condition, including the emotional strain of monitoring, worries about complications, and potential impact on relationships [4]. PIR involves hesitating or refusing to start insulin treatment, which can delay necessary care [5]. FOH is a state of anxiety and discomfort, which is common in diabetic patients, especially those with hypoglycemia or at risk of developing it [6]. These mental health and diabetes-related psychological factors can decrease participation in self-management, lead to suboptimal glycemic control, lower quality of life, and increase the risk of complications and early mortality [7].

Much of the existing literature examines DRD across combined type 1 diabetes mellitus (T1DM) and type 2 diabetes mellitus (T2DM) populations, which may obscure important differences. Distress in T1DM more often centres on fear of hypoglycaemia, whereas in T2DM it is more commonly associated with guilt or shame linked to lifestyle factors [8]. A systematic review found the overall prevalence of DRD to be 36% in patients with type 2 diabetes mellitus (T2DM) [8]. Similarly, a meta-analysis in China found DRD in 50.5% of patients with T2DM [9]. The psychological strain of DRD extends beyond a patient’s mental well-being, adversely affecting their diabetes management, overall quality of life, and glycemic control [10]. DRD can impair self-management behaviours, such as medication adherence, healthy eating, and physical activity, accelerating disease progression [11]. PIR, most commonly described in people with T2DM, involves hesitation or refusal to initiate insulin therapy, often leading to delays in necessary treatment [5]. Individuals with T2DM experiencing PIR often perceive the transition to insulin as a personal failure, attributing it to inadequate self-management and subsequently experiencing guilt and remorse [12, 13]. FOH is manifested as the fear of the negative impact of hypoglycemia on health and life, fear of an emergency, fear of losing self-control, and the onset of behavioral or cognitive disorders [14].

Stigma has become a significant barrier to self-management in patients with diabetes [15]. Recent evidence suggests that diabetes-related stigma differs between T1DM and T2DM. In adults, reported prevalence ranges from 52% to 78% in T1DM and 12%–70% in T2DM, with youth studies showing stigma is particularly common in T1DM. Differences may reflect clinical and social factors, such as visible insulin use in T1DM versus lifestyle-related blame in T2DM, influencing psychosocial functioning and self-care [15]. Stigma associated with diabetes elevates the risk of mental health issues, such as psychological distress and depression [16], significantly impacting the overall biopsychosocial well-being of affected individuals [17]. Despite the significant impact of mental health on diabetes care, psychological issues are often overlooked and undertreated in clinical practice. Routine screening using validated tools such as the Patient Health Questionnaire-9 (PHQ-9) for depression and the Diabetes Distress Scale (DDS) for diabetes-related distress has been shown to improve both psychological well-being and clinical outcomes in individuals with T2DM [18]. Several novel integrated care models have emerged in recent years, demonstrating that combining psychiatric and diabetes care can lead to improvements in both mental and physical health outcomes [19].

The primary purpose of this review is to highlight the prevalence of mental health issues in people with diabetes and the impact if left untreated. It also examines the bidirectional interaction between these conditions, and discusses the implications for clinical practice, with a focus on integrated treatment approaches that address both physical and mental health. Crucially, this review stresses the necessity for increased awareness among both physicians and psychiatrists regarding the significant interplay between diabetes and mental health.

## Prevalence of mental health disorders in diabetes and their bidirectional relationship

The bidirectional relationship between diabetes and mental health disorders is well documented [1, 20]. This relationship is influenced by biological mechanisms, including chronic low-grade inflammation, dysregulation of the hypothalamic–pituitary–adrenal (HPA) axis, and impaired glucose metabolism, as well as behavioural factors such as sedentary lifestyle, unhealthy diet, poor treatment adherence, and reduced sleep quality. Individuals suffering from serious mental disorders, such as schizophrenia and bipolar disorder, are more likely to develop T2DM than the general population [21]. Depression is associated with a 37% higher risk of T2DM [22]. Mental health disorders are highly prevalent among individuals with diabetes, significantly impacting disease management and quality of life (Table 1). Depression is particularly common, with a pooled prevalence of 37.8% reported among Chinese T2DM patient [9]. In Romania, depression affects 34.3% of diabetic individuals, with even higher rates (up to 61.8%) seen in Iranian populations [23, 24]. Anxiety is also prevalent, affecting 28.9% of Chinese and 44.9% of Romanian diabetic patients, with higher rates typically observed in women. Diabulimia (intentional insulin restriction to control weight) poses a serious risk, particularly in females with type 1 diabetes. One study found a prevalence of 10.3% for insulin omission, with up to 31% of females reporting some engagement in this behavior and 9% doing so regularly [25–27]. Cognitive impairment is another concern, particularly among older adults with T2DM. Studies have reported mild cognitive dysfunction in 38.9% to 69% of patients, with depression and anxiety significantly associated with cognitive decline [23, 28].

**Table 1:** Prevalence of mental health disorders in diabetes

Mental health/Psychological condition	Prevalence	Notes/Population Details	References
Depression	37.8%- 61.8%	More common among younger individuals and those with higher education; figures combine T1DM and T2DM populations.	[9, 23, 24, 32]
Anxiety	28.9%- 48.9%	Diabetes is associated with 41% higher risk of anxiety disorders.	[9, 23, 33]
Diabetes-related distress	36%–50.5%	Higher prevalence among women and those with comorbid depressive symptoms; 36% in multinational sample, 50.5% in T2DM China-specific sample.	[8, 9]
Eating disorders	24% among people with T1DM; 31% of females report some insulin-omission behaviour, with 9% reporting frequent omission	Higher prevalence in female participants; behaviours such as insulin restriction are often linked to weight concerns and glycemic control.	[25–27, 34]
Cognitive Impairment	38.9%- 69%	Cognitive Impairment is more prevalent in T2DM patients (median age, 58 years).	[23, 28]
Schizophrenia	13% (coexistence rates)	The trend of coexistence of the two illnesses was relatively stable over eight years.	[35]

Mental health problems, such as depression, further reduce cognitive function and motivation, impairing diabetes management by 76% [29]. The coexistence of diabetes and mental disorders is linked to common biological mechanisms like inflammation, HPA axis dysfunction, and altered glucose metabolism [30]. Effective care requires addressing both physical and mental health. Integrating mental health care into diabetes management improves treatment adherence, glycemic control, and quality of life. Cognitive-Behavioural Therapy (CBT) and collaborative care approaches have proven beneficial, highlighting the need for routine screening for depression, anxiety, and diabetes-related distress in adults with T2DM [31]. These and other strategies including mindfulness-based interventions, pharmacological treatments, lifestyle changes, peer support, and technology assisted care are examined in detail later in this review, with attention to their effectiveness and applicability.

### The impact of untreated mental health issues in diabetes

Diabetes and untreated mental health issues often co-occur, leading to significant adverse impact on both health and the economy. Research shows that individuals with both diabetes and mental health disorders face increased healthcare costs and hospitalization rates, greater risk of medical complications, and reduced quality of life, among other adversities.

- *Increased Healthcare Costs and Hospitalization Rates*
  The healthcare costs for individuals with diabetes and untreated mental health conditions are significantly higher due to more frequent use of healthcare services such as hospital stays, ER visits, and outpatient consultations. In a large health insurance cohort, individuals with depression had healthcare costs that were approximately 1.49 times higher than those without depressive symptoms, after adjusting for key sociodemographic and clinical factors [36]. Moreover, untreated mental health issues are associated with higher hospitalization rates, particularly for complications like diabetic ketoacidosis and cardiovascular events [37, 38]. Individuals with depression are twice as likely to be hospitalized for diabetes-related issues, stressing the need for integrated care [39].
  A study using data from the 2004–2011 Medical Expenditure Panel Survey, a nationally representative estimate of healthcare expenditures in the United States, showed that the average annual medical cost for patients with diabetes but no depression was $10,016, while for those with symptomatic depression, it was $20,105, effectively doubling the cost burden [40]. In England, a longitudinal matched-cohort study found that average annual healthcare costs were £1930 higher for people with T2DM, and severe mental illness (SMI) compared to those with T2DM alone [41]. These higher costs were primarily driven by mental health and non-mental health-related hospital admissions. It has also been reported that approximately £1.8 billion of additional costs to the NHS can be attributed to poor mental health in people with T2DM [42].
- *Increased Risk of Complications and Mortality*
  Untreated mental health conditions, particularly depression, are linked to a higher risk of diabetes-related complications such as retinopathy, neuropathy, and nephropathy. Poor glycemic control, which is often a result of inadequate self-management due to mental health struggles, contributes to these complications [43]. A meta-analysis found that people with untreated mental health conditions are 36% more likely to develop diabetes-related complications [44]. Additionally, the risk of death is two to three times higher for individuals with both T2DM and untreated depression compared to those without mental health issues, primarily due to poor treatment adherence and cardiovascular complications [45].
- *Reduced Adherence to Treatment and Quality of Life*
  Mental health conditions such as depression and diabetes-related distress significantly reduce treatment adherence [46]. This includes poor medication adherence, unhealthy eating habits, and low physical activity levels, all of which worsen glycemic control and increase the risk of complications [47]. Non-adherence is four times more common in individuals with depression [29]. The combination of diabetes and untreated mental health issues also leads to a significantly lower health-related quality of life (HRQoL). Systematic reviews which have synthesized findings from studies utilizing various HRQoL measures such as the SF-12, report that individuals with both conditions experience 50–60% lower HRQoL scores compared to those without mental health conditions [48]. The emotional strain of managing both conditions makes integrated mental health care essential for improving overall well-being.
- *Increased Emergency Room Visits and Cognitive Decline*
  Untreated mental health conditions in diabetes patients often lead to more frequent ER visits for acute complications, such as Hypoglycemia and diabetic ketoacidosis. Individuals with both diabetes and unmet psychological needs are three times more likely to require emergency care [39]. Additionally, untreated mental health conditions increase the risk of cognitive decline and dementia, further complicating diabetes management [49]. Anxiety and depression contribute to cognitive impairment through metabolic imbalance and chronic inflammation, making it harder for individuals to adhere to self-care guidelines [50].

### Management of mental health conditions in diabetes

Addressing mental health concerns in individuals with diabetes is essential for improving both psychological well-being and diabetes-specific clinical outcomes. Evidence-based interventions such as psychological therapies, medication, and lifestyle changes have been shown to reduce the distress associated with diabetes, anxiety, and depression. Key guidelines from the US (American Diabetes Association) [51], Canada (Diabetes Canada) [52], and the UK (National institute of clinical excellence & Diabetes UK) [53, 54], stress the importance of integrating mental health support into diabetes care. Regular screening for mental health issues like anxiety, depression, and diabetes-related distress, both at diagnosis and during follow-up, is a central recommendation.

While these guidelines provide a strong framework, their application often requires adaptation for culturally and ethnically diverse populations, both within high-income countries and in low- and middle-income countries (LMICs). Within high-income settings, differences in cultural beliefs, language, stigma, and health literacy can influence engagement with care, while in LMICs additional challenges include resource limitations and the role of traditional health practices [55, 56]. Systematic reviews highlight the need for contextualized content and delivery of guidelines in LMICs to improve outcomes [57]. Additionally, studies have explored the cultural adaptation of diabetes self-management and prevention programs to increase relevance and comprehensibility in such settings [58, 59].

Below is a summary of key management strategies:

- *Screening and Early Detection*
  Early screening for mental health disorders, including depression, anxiety, and diabetes-related distress, is critical. The American Diabetes Association recommends that mental health assessments be conducted at the time of diagnosis, during annual reviews, when complications arise, or when there are significant changes in disease status or treatment [60]. Validated tools such as the PHQ-9 for depression [61] and the Generalized Anxiety Disorder-7 scale (GAD-7) for anxiety [62] are commonly used. A PHQ-9 score ≥ 10 or GAD-7 score ≥ 10 typically indicates moderate symptoms and warrants further evaluation or referral. The DDS [63] is recommended when diabetes-specific distress is suspected; scores ≥ 3 on any subscale suggest clinically significant distress, prompting psychosocial support or psychological intervention.
- *Psychological Interventions*
  Cognitive-Behavioural Therapy (CBT), Mindfulness-Based Stress Reduction (MBSR), and Problem-Solving Therapy (PST) are evidence-based psychological interventions shown to benefit individuals with diabetes. While broadly beneficial across different diabetes types and age groups, their specific application and focus may be tailored. For instance, CBT interventions are often adapted to address unique concerns in individuals with Type 1 Diabetes, such as fear of hypoglycemia and adherence challenges in adolescents, whereas in T2DM, they may focus more on lifestyle modifications, weight management, and comorbidity management. A systematic review and meta-analysis in pateints with T2DM found significant improvements in both depressive symptoms and glycemic control [64]. MBSR enhances emotional regulation and reduces stress, contributing to better diabetes self-care and lower HbA1c levels in adults with T2DM [65]. PST focuses on developing practical skills to manage the emotional and problem-solving demands of living with diabetes [66]. The American Association of Diabetes Educators [67] has identified problem solving as one of seven core diabetes self-management behaviours.
- *Pharmacological Treatment*
  Pharmacological interventions, such as antidepressants, are often used to manage depression and anxiety in people with diabetes. Serotonin-norepinephrine reuptake inhibitors (SNRIs) and selective serotonin reuptake inhibitors (SSRIs) are commonly prescribed. A meta-analytic review of literature found that antidepressants can improve glycemic control by enhancing self-care adherence. However, clinicians should monitor potential side effects, as some antidepressants may impact glucose metabolism [68].
- *Integrated Collaborative Care Models*
  Integrated care approaches, involving a multidisciplinary team of healthcare providers, have proven effective in managing both mental and physical health in diabetes [69]. Collaborative care, which includes primary care physicians, endocrinologists, and mental health specialists, leads to better glycemic control and reduced healthcare costs [70, 71]. The comorbidity of depression and diabetes is associated with increased disability, poorer clinical outcomes, and premature mortality, yet mental health remains insufficiently addressed in many chronic disease care models [72]. It is established that integrated care results in improved health outcomes and lower healthcare expenses, as it ensures that mental and physical health are managed simultaneously [70].
  However, disparities in access to integrated care persist, with significant challenges observed, particularly in rural areas and among low-income populations in low- and middle-income countries (LMICs) where there is a scarcity of specialized mental health providers, geographical barriers to clinics, and systemic inequities in healthcare funding and infrastructure [73]. Addressing these challenges is crucial for achieving equitable health outcomes for all individuals with diabetes globally.
- *Lifestyle Interventions and Physical Activity*
  Lifestyle changes, particularly physical activity, are essential for managing diabetes-related mental health problems. Lifestyle changes, particularly physical activity, are essential for managing diabetes-related mental health problems. Many studies have emphasised the health benefits of physical activity, with growing evidence demonstrating positive effects of exercise specifically in individuals with T2DM [74]. Within an integrated care framework, it is recommended to refer patients to allied health professionals, such as exercise physiologists or physical therapists, who can provide tailored exercise prescriptions and ongoing support to help individuals effectively implement and sustain these vital lifestyle changes [60].
- *Social Support and Peer Support Groups*
  Social support is vital for managing mental health in people with diabetes. For individuals with T1DM, peer support and social networks can provide emotional encouragement, practical guidance, and a sense of connection, which may help reduce diabetes-related distress and support effective self-management [75]. In T2DM, higher levels of social support are associated with improved clinical outcomes, healthier self-management behaviours, and adoption of beneficial lifestyle activities [76]. Peer support programs enhance glycemic control and lower mental distress, highlighting the importance of supportive networks for managing T2DM [77].
- *Technology-Assisted Interventions*
  Technology-based interventions, such as telehealth CBT augmented with mobile health applications, has demonstrated improvement in T2DM management and distress [78]. There is evidence of significant reductions in depression, anxiety, stress, and glucose levels, along with an increase in the frequency of glucose self-testing, among diabetic participants in behavioural telehealth programmes [79]. Furthermore, there is evidence that behavioural activation therapy (BAT) delivered via telemedicine is more effective than in-person delivery in reducing mean A1c levels in adults with T2DM, indicating that telemedicine-based BAT is a viable treatment option for this population [80]. However, the effectiveness and reach of these technology-based approaches are often limited by significant digital equity and accessibility issues in lower-resource settings, including inadequate internet infrastructure, lack of affordable devices, and low digital literacy among certain populations, particularly in rural areas and low- and middle-income countries [81].

Table 2 outlines the essential roles of endocrinologists and physicians in addressing the mental health needs of patients with diabetes. It emphasizes the importance of proactive screening, collaborative care, and tailored behavioural support, alongside patient education, and regular monitoring of both psychotropic and antidiabetic medication.

**Table 2:** Role of endocrinologists and physicians in addressing the mental health needs of diabetes patients

Role	Details
Screening for mental health issues	A proactive and routine approach to screening, tailored across the life course and care settings, supports early diagnosis and timely treatment. Building staff skills in recognizing risk and responding appropriately is essential to prevent delays in intervention in T2DM [82].
Collaborative care coordination	Facilitate regular follow-up and communication between care team members to support individualised, treat-to-target goals in both mental health and Diabetes management [70].
Behavioural activation and goal setting	Support behaviour change by encouraging pleasurable activities, structured goal-setting, and problem-solving to enhance treatment adherence and mood [70].
Creating a psychologically safe consultation space	Practitioners should provide an open, empathetic environment to help patients with T1DM feel safe discussing emotional concerns and changes in wellbeing [83].
Patient education and support	Educate patients on the connection between diabetes and mental health, encourage self-care, and provide resources for managing distress [84].
Medication management	Ensure regular monitoring of both psychotropic and antidiabetic medications, minimizing metabolic risk where possible. Encourage coordination between psychiatric and medical teams [85].
Training and workforce development	Invest in training multidisciplinary staff to integrate mental health competencies into diabetes care. System-level strategies should include continuous professional development, supervision structures, and workforce planning to ensure sustainable, integrated care delivery for patients with T1DM and T2DM [86].

### Future directions

Despite significant advancements in understanding and managing the intersection of diabetes and mental health, several critical gaps remain, highlighting priority areas for future investigation. Future research and service development should place greater emphasis on tailored psychological care that recognises the distinct lived experiences and care needs associated with T1DM and T2DM, rather than assuming a uniform model of psychological comorbidity across diabetes types. For instance, fear of hypoglycaemia and treatment adherence challenges are more prominent in T1DM, whereas lifestyle-related distress, stigma, and comorbidity management are often central in T2DM. Understanding these distinctions is essential to designing effective, personalised care strategies.

There is also an urgent need for robust research on the impact of digital interventions in lower-resource settings, particularly in LMICs. This includes evaluating their efficacy, scalability, and long-term sustainability, while addressing digital equity and accessibility issues. Comprehensive cost-effectiveness analyses of integrated care models are crucial, particularly those assessing long-term outcomes across diverse healthcare systems and patient populations. Future studies should explore the effectiveness of combining different psychological interventions and develop standardised outcome measures that capture both mental and metabolic health improvements.

By prioritising tailored, context-specific approaches and recognising the differences in experiences of psychological comorbidity between T1DM and T2DM, future research can optimise care strategies and improve equitable health outcomes for individuals living with diabetes worldwide.

## Conclusion

The interconnection between diabetes and mental health underscores the urgent need for comprehensive, person-centered care. Routine screening, evidence-based psychological and pharmacological interventions, and integrated service delivery models are vital to improving outcomes. Policymakers and healthcare providers must prioritize integrated approaches to effectively address the dual burden of mental and physical illness in diabetes, ultimately enhancing well-being and reducing healthcare complexities for affected individuals globally.

## Data Availability

No datasets were generated or analysed during the current study.
